# Role of NR1D1 in Bisphenol A-Induced Anxiety-like Behavior and Inflammation in Zebrafish Larvae

**DOI:** 10.3390/toxics13060449

**Published:** 2025-05-28

**Authors:** Mingjun Wu, Pinyi Chen, Yuting Wang, Xinwei Wang, Yuqianrui Bao, Liqiao Fan, Yuxiao Rao, Xiaoyao Song, Jie Zhang

**Affiliations:** School of Public Health, Medical College of Soochow University, Suzhou 215127, China; 18081873307@163.com (M.W.); chenpy0219@163.com (P.C.); 13206462812@163.com (Y.W.); w19853146632@163.com (X.W.); 18013617223@163.com (Y.B.); luhe20040130@gmail.com (L.F.); 2430504082@stu.suda.edu.cn (Y.R.)

**Keywords:** bisphenol A (BPA), NR1D1, anxiety-like behavior, neuroinflammation, zebrafish larvae

## Abstract

Bisphenol A (BPA) is a widespread environmental endocrine disruptor with significant neurodevelopmental and behavioral risks. The present study explored the role of the circadian clock protein NR1D1 in mediating BPA-induced anxiety-like behavior and brain inflammation early in life. Zebrafish embryos exposed to BPA exhibited anxiety-like behavior characterized by altered motor activity patterns. Notably, BPA exposure suppressed the expression of the circadian clock gene nr1d1, accompanied by increased transcriptional and protein levels of pro-inflammatory cytokines, including IL-6, IL-1β, and TNF-α. These changes created a pro-inflammatory microenvironment that disrupted dopamine system homeostasis, contributing to the observed behavioral abnormalities. Activation of NR1D1 using GSK effectively reversed BPA-induced inflammatory responses and restored normal dopamine levels and behavioral phenotypes. These findings highlight NR1D1 as a critical regulator linking circadian rhythm disruption, neuroinflammation, and dopaminergic dysfunction to anxiety-like behavior. This study provides novel insights into the mechanisms underlying BPA-induced neurotoxicity and identifies NR1D1 as a potential therapeutic target for mitigating the adverse effects of early-life BPA exposure.

## 1. Introduction

Bisphenol A (BPA) is a widely utilized endocrine-disrupting chemical (EDC) that is employed in the internal coating of plastic cups, infant feeding bottles, and food and beverage cans [1,2,3]. A substantial body of evidence has indicated a robust correlation between early life exposure to EDCs, including BPA, and the emergence of anxiety disorders. Anxiety disorders are characterized by persistent and excessive fear or worry. They are among the most prevalent and disabling mental health conditions in the world, posing a significant public health burden [4,5]. There is a correlation between prenatal exposure to BPA and the development of anxiety-like behavior, mood disorders, and impaired exploratory behavior in both animals and humans [6,7]. The extant evidence indicates that there is a positive association between prenatal exposure to BPA and the development of anxiety disorders in adolescence [8]. For instance, research has demonstrated that early-life exposure to bisphenol A (BPA) in zebrafish larvae induces anxiety-like behaviors and decreases exploratory tendencies in rodent models [9,10]. However, the precise mechanisms underlying BPA-induced anxiety-like behaviors remain uncertain.

Circadian rhythms are defined as biological oscillations that occur in cycles of approximately 24 h. Recent studies have demonstrated a correlation between circadian rhythm disruption and a number of psychiatric disorders, including anxiety disorders and depression [11,12]. Mice exposed to low doses of BPA during pregnancy have been shown to exhibit significant impairments in circadian activity, social behavior, and related neurological functions in adulthood [13,14]. Furthermore, genetic studies have identified a correlation between polymorphisms in clock genes that regulate circadian rhythms and an increased risk of developing anxiety disorders [15]. The experimental model suggests that circadian rhythm disorders (e.g., sleep disorders) can induce anxiety-like behaviors and neuroinflammation by altering the expression of clock genes [16]. In summary, the hypothesis is proposed that circadian rhythm disruption may act as a key mechanism for the neurotoxic effects of BPA.

The Nuclear Receptor Subfamily 1 Group D Member 1 (NR1D1) has been identified as a central component of the circadian clock system, playing a pivotal role in maintaining circadian rhythm homeostasis. NR1D1 functions as a transcriptional repressor by recruiting nuclear receptor co-repressors and histone deacetylase 3 to its target genes [17]. Interestingly, BPA has been shown to alter circadian rhythms by modulating the activity of hypothalamic progenitor cells through estrogen and androgen receptors [18]. Furthermore, the presence of binding sites for the estrogen receptor (ESR1) and androgen receptor (AR) on the NR1D1 promoter was indicated using the website https://jaspar.genereg.net/ (accessed on 22 May 2022), suggesting potential regulatory interactions. Based on these findings, we hypothesize that BPA disrupts nr1d1 transcriptional activity by interfering with estrogen and androgen receptor signaling, ultimately leading to anxiety-like behavior.

In this study, we explored the effects of BPA on tactile behavior and spontaneous locomotion in zebrafish larvae. We employed behavioral experiments to confirm that BPA induces anxiety and disrupts the circadian rhythm. The present study employed a comprehensive suite of bioinformatics analyses to elucidate the molecular mechanisms underlying the effects of BPA on gene expression, with a particular focus on the circadian rhythm, dopaminergic neuron differentiation, and inflammatory responses. In particular, we explored the possible role of nr1d1 in mediating BPA-induced anxiety-like behaviors by examining downstream regulatory targets of nr1d1 and related molecular pathways. This study provides novel insights into the mechanisms of anxiety development triggered by exposure to BPA early in life and identifies potential therapeutic targets for anxiety disorders.

## 2. Materials and Methods

### 2.1. Chemical and Reagent Preparation

BPA powder was purchased from Sigma-Aldrich (99%, St Louis, MO, USA). A stock solution of BPA was prepared by dissolving the compound dimethyl sulfoxide (DMSO, Sigma-Aldrich, St Louis, MO, USA) and diluted with E3 medium to achieve the desired treatment concentrations. DEPC-treated water (Beyotime, Nanjing, China) was used for cleaning zebrafish embryos and diluting cDNA. The NR1D1 agonist GSK4112 (GSK) was purchased from Sigma-Aldrich (St Louis, MO, USA). Additional reagents included TRIzol reagent (Beyotime) and the RevertAid First Strand cDNA Synthesis Kit (Thermo Fisher Scientific, Waltham, MA, USA).

### 2.2. BPA, GSK, and BPA + GSK Exposure of Zebrafish Embryos

**Figure 1:**
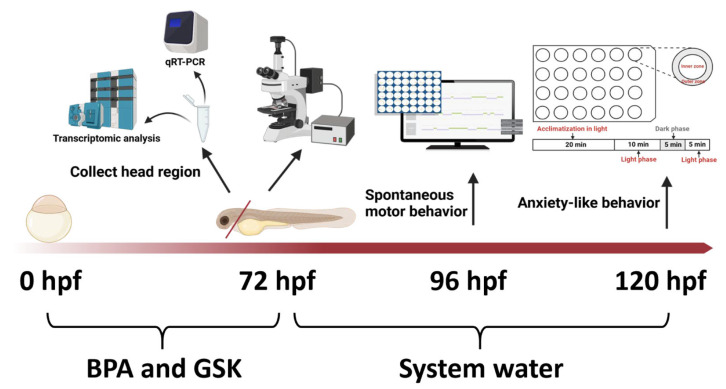
A workflow of the present study design.

The workflow of the present research is in Figure 1. The zebrafish (Danio rerio) were obtained from the Zebrafish Research Center at Soochow University (Soochow, China). The fish were maintained under standard laboratory conditions at 28.5 °C with a 14 h light/10 h dark (LD) cycle and were fed three times daily. The healthy zebrafish embryos were randomly distributed into 6-well plates, with 30 embryos per well. Four experimental groups were established. The solvent control (DMSO concentration: 0.1%) was prepared by diluting 3 μL of DMSO in 3 mL of E3 water. The GSK solution was diluted to a concentration of 1 μmol/L. The solution of bisphenol A (BPA) was diluted to a concentration of 25 μmol/L, which was designated as the exposure concentration. The BPA + GSK group was treated with a combination of the two solutions. The exposure period for BPA and GSK is 0–72 hpf. Each experimental group comprised three replicates. The plates were incubated at 28.5 ± 0.5 °C with a 14 h light/10 h dark cycle. The embryos were washed with DEPC water (Beyotime) and the cDNA was diluted with the same solution.

### 2.3. Locomotor Behavioral Analysis

Anxiety-like behavior in zebrafish was evaluated using a thigmotaxis test. This protocol was adopted subsequent to Schnörr et al. (2012), with dwelling time in the central circle constituting the primary behavioral metric [19]. The zebrafish larvae were exposed to the BPA until 72 h post-fertilization (hpf), after which they were cultured in system water until 120 hpf. For each group, six larvae were placed in individual wells of a 24-well plate with the system water level adjusted to allow for free movement. The central area was defined as the region situated at a distance of 4 mm from the well walls. The plate was placed in a zebrafish box for behavioral tracking, and the 95 min experimental protocol consists of a 10 min adaptation period followed by 40 min continuous light and three cycles of 10 min light and 5 min darkness. The time spent by the larvae in the central region during the continuous light and alternating light–dark phases was recorded to evaluate their thigmotaxis and anxiety-like behavior [20].

The spontaneously occurring movements of zebrafish larvae governed by circadian activity were analyzed under constant darkness conditions. The zebrafish larvae were exposed to the BPA until 72 h post-fertilization (hpf) and, subsequently, the larvae were then transferred to system water and cultured until 96 hpf. Subsequently, a fish was placed in each of the 48 wells of the plate, with 12 fish allocated to each group. System water was then added to each well until the water level reached the rim, thus allowing the fish to move freely within the wells. The 48-well plate was subsequently introduced into the DanioVision system. The spontaneous behavior of zebrafish larvae was observed from 96 hpf under constant darkness conditions and recorded continuously for five days. The parameters that were set included position (X, Y), length-to-width ratio, minimum detection threshold, number of wells, background color, minimum activity threshold, and inactivity threshold. During this period, the distance traveled by juveniles was recorded and analyzed at ten-minute intervals for a period of five days using EthoVision XT 10.0 software. The analysis of circadian rhythm parameters was conducted utilizing the BioDare2 platform (https://biodare2.ed.ac.uk/, accessed on 26 January 2021) [21]. The platform utilized the MFourFit algorithm, in conjunction with pertinent parameters, to calculate the period, phase and amplitude of spontaneous locomotor rhythms. These rhythm parameters were then analyzed in comparison with control groups (the utilization of solely the middle three days of data), thereby enabling a systematic assessment of circadian rhythm disturbances in BPA-exposed zebrafish larvae [22].

### 2.4. Transcriptomic Analysis

At 96 hpf, samples of zebrafish larvae were collected for total RNA extraction. The quality of the RNA was evaluated, and total RNA was extracted using the mirVana miRNA Isolation Kit (Thermo Fisher Scientific, Waltham, MA, USA) following the manufacturer’s protocol. RNA integrity was evaluated using the Agilent 2100 Bioanalyzer (Agilent Technologies, Santa Clara, CA, USA). The samples with RNA integrity number (RIN) ≥ 7 were subjected to the subsequent analysis. The libraries were constructed using the TruSeq Stranded mRNA LTSample Prep Kit (Illumina, San Diego, CA, USA) according to the manufacturer’s instructions. Then these libraries were sequenced on the Illumina sequencing platform (HiSeqTM 2500 or Illumina HiSeq X Ten) (Illumina, San Diego, CA, USA) and 125 bp/150 bp paired-end reads were generated. Differentially expressed genes were identified based on a *p* value < 0.05 and fold change (|FC|) > 2 or |FC| < 0.5. Subsequently, the genes were subjected to analysis with Gene Ontology (GO) and Kyoto Encyclopedia of Genes and Genomes (KEGG) databases.

### 2.5. RT-qPCR

Total RNA was extracted from zebrafish larvae by using TRIzol reagent (Beyotime, Shanghai, China). RNA concentration and optical density (OD) values were measured with a NanoDrop spectrophotometer (ThermoFisher) ensuring that the A260/A280 ratios of all samples fell within the range of 1.8 to 2.0. cDNA synthesis was performed using a reverse transcription kit (ThermoFisher). Quantitative PCR was conducted with a QuantStudio6 system (ThermoFisher) utilizing SYBR Green Master Mix (ROX). The PCR protocol involved an initial step of 120 s at 95 °C, followed by 45 cycles of 95 °C for 20 s and 60 °C for 40 s. Gene expression levels were quantified in triplicate for each treatment, with β-actin serving as the housekeeping gene. Relative gene expression changes were calculated using the 2^−ΔΔCT^ method. The primer sequences are presented in Table S1.

### 2.6. Astrocyte Function Detection

Healthy Tg (gfap:EGFP) zebrafish (provide by Department of Medical Genetics, School of Basic Medical Sciences, Suzhou Medical College, Soochow University) embryos were randomly distributed into a six-well plate, with 30 embryos per well. The plate was divided into four groups, designated as control, GSK, BPA, and BPA + GSK. The embryos were incubated under standard conditions at 28.5 ± 0.5 °C with a 14 h light/10 h dark cycle. During the incubation period, the embryos were monitored meticulously, and any that did not survive were promptly removed. The culture medium was replaced on a daily basis until the 72 hpf stage. Following a period of 72 hpf, the embryos were transferred to a solution of zebrafish system water and maintained until 96 hpf. At 96 hpf, ten transgenic zebrafish larvae from each group were selected for imaging. Fluorescent images were captured under a fluorescence microscope with a constant camera setting. The microscope and the camera setting are as follows: microscope: Nikon (SZ61, Tokyo, Japan); zoom ratio of 6.7:1 (0.67×–4.5×); objective magnification: CFI 10×/22; resolution approx. 1.5 μm; low-intensity LED light source (<100 lx); field of view number (FN): 22 mm. The fluorescence intensity was subsequently analyzed using the Image J (version 1.53).

### 2.7. Inflammation-Related Factor Detection

At 96 hpf, larvae from each treatment group were homogenized, with three replicates in each treatment. Enzyme-linked immunosorbent assay (ELISA) kits (Affandi, Shanghai, China) were employed to quantify the levels of tumor necrosis factor-alpha (TNF-α), interleukin-1β (IL-1β), and interleukin-6 (IL-6) in the zebrafish larvae tissue. The assays were conducted in accordance with the manufacturer’s instructions, with the detection ranges for TNF-α, IL-1β, and IL-6 being 10 ng/L to 360 ng/L, 3 ng/L to 90 ng/L, and 0.5 ng/L to 12 ng/L, respectively. Furthermore, the influence of distinct treatments on neutrophil migration was investigated using Tg (lyz: EGFP) zebrafish larvae. The fluorescence intensity in the head region was quantified using ImageJ software.

### 2.8. Dopamine, DOPA, and Norepinephrine Content Detection

At 96 h post-fertilization (hpf), samples were collected from each of the experimental groups (60 samples in total, with four replicates). The samples were stored at −80 °C and then homogenized in 50 μL of water using an MP homogenizer (MP Biomedicals, Santa Ana, CA, USA). Subsequently, the samples were vortexed 60 s, after which 200 μL of methanol: acetonitrile (1:1, *v*/*v*) was added. The mixture was then vortexed again for a further 60 s and subjected to low-temperature sonication for a 90 s of with this process being repeated twice. Following a one-hour incubation period at −20 °C to precipitate proteins, the samples were subjected to centrifugation at 14,000 rpm for 20 min at 4 °C. The resulting supernatant was then collected, freeze-dried, and stored at −80 °C. For analysis, an Agilent 1290 Infinity LC ultra-high-performance liquid chromatography (UHPLC, Agilent, Santa, CA, USA) system was used. The mobile phases employed were as follows: (A) 0.1% formic acid in 25 mM ammonium formate aqueous solution and (B) 0.1% formic acid in acetonitrile. The samples were placed in the autosampler at 4 °C, with the column maintained at 45 °C, a flow rate of 300 μL/min, and an injection volume of 2 μL. The gradient was as follows: from 0 to 18 min, B increased linearly from 90% to 40%; from 18 to 18.1 min, B increased linearly from 40% to 90%; and from 18.1 to 23 min, B remained at 90%. A 5500 Q-Trap mass spectrometer (AB SCIEX, Woodlands Central Industrial Estate, Singapore) was employed for mass spectrometry analysis in positive ion mode. The electrospray ionization (ESI) source conditions for the 5500 Q-Trap mass spectrometer (SCIEX, Framingham, MA, USA) were as follows: source temperature 450 °C, ion source gas 1 (gas 1) 60, ion source gas 2 (gas 2) 60, curtain gas (CUR) 30, ion spray voltage floating (ISVF) 5000 V (AB Sciex Pte. Ltd., Singapore).

### 2.9. Data Analysis

The data were processed and visualized using Excel and GraphPad Prism 8 software, as appropriate for the analysis. The quantification of GFP fluorescence was conducted in accordance with the methodology outlined in the reference [20]. The resulting data were presented as mean ± standard deviation, and statistical analysis was performed using SPSS 22.0. A two-way analysis of variance (ANOVA) was employed to examine the proportion of time spent by zebrafish larvae in the central area under alternating light and dark conditions. One-way ANOVA was applied to evaluate the proportion of time spent in the central area during continuous light exposure and to assess the activity rhythm metrics of zebrafish larvae. DEGs were identified using the DESeq (2012) R package functions estimate Size Factors and nbinomTest. *p*-value < 0.05 and |FC| > 2 or |FC| < 0.5 were set as the threshold for significantly differential expression. Hierarchical cluster analysis of DEGs was performed to explore gene expression patterns. GO enrichment and KEGG pathway enrichment analysis of DEGs were respectively performed using R based on the hypergeometric distribution. The results of the qPCR were analyzed using *t*-tests, with a significance level set at α = 0.05. A *p*-value of less than 0.05 was deemed to be statistically significant.

## 3. Results

### 3.1. Transcriptomic Profiling

**Figure 2:**
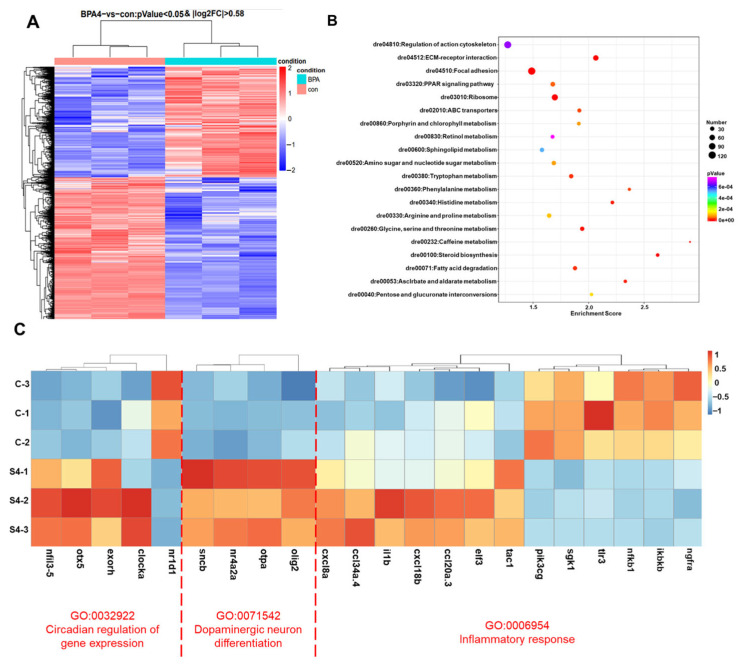
(**A**) The heatmap illustrates the overall distribution of differentially expressed genes between the BPA-exposed and control groups. The color intensity represents the level of gene expression (normalized counts). Data are presented as mean ± standard deviation. & indicates a significant difference from the con group, with *p* < 0.05 and, |log2FC| > 0.58. (**B**) KEGG pathway enrichment analysis of DEGs, highlighting the top 20 significantly enriched pathways (*p* < 0.05). (**C**) Cluster analysis of DEGs under three terms. c-1, c-2, and c-3 represent the biological replicates from the control group, while S4-1, S4-2, S4-3 represent three biological replicates from the BPA-exposed group.

In order to conduct an investigation into the gene expression changes that occur in zebrafish larvae when exposed to 25 μmol/L BPA, a transcriptomic analysis was performed. This analysis was undertaken with the objective of identifying any differentially expressed genes (DEGs) that may be present. The heatmap displays the expression profile of various genes associated with BPA exposure. A total of 8366 differentially expressed genes (DEGs) were identified, of which 3444 were found to be up-regulated and 4922 down-regulated in comparison to the control group (Figure 2A and Figure S1). GO screening and KEGG enrichment analysis revealed significant alterations in genes associated with circadian regulation, dopaminergic neuronal differentiation, and inflammatory responses (Figure 2B). Among the circadian clock genes, *nr1d1* was significantly down-regulated, while *clocka* and pineal function genes (e.g., *exoh*, *otx5*) were up-regulated. Furthermore, an up-regulation of dopaminergic regulatory genes was observed, including nuclear receptor 4A subfamily (*nr4a2a*) and oligodendrocyte transcription factor 2 (*olig2*). Inflammatory-related genes, including chemokine family (*cl20a.3, cl34a.4, cxcl8a, cxcl18b*) and interleukin 1β (*il1β*)-related genes, were notably elevated, while genes such as Toll-like receptor 3 (*tlr3*), nerve growth factor receptor A (*ngfra*), serum/glucocorticoid-regulated kinase 1 (*sgk1*), and nuclear factor kappa B subunit 1 (*nf-κb1*) genes demonstrated a downward trend (Figure 2C). These findings suggest that BPA disrupts circadian rhythms and induces inflammatory responses, with *nr1d1* emerging as a key target. Further investigation focused on the role of *nr1d1* in BPA-induced anxiety-like behavior.

### 3.2. BPA Alters the Thigmotactic Behavior in Zebrafish Larvae

**Figure 3:**
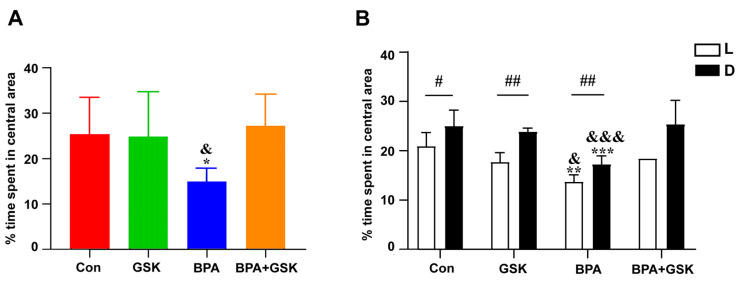
(**A**) Percentage of time spent in the central zone by zebrafish larvae under continuous illumination across different treatment groups. (**B**) Percentage of time spent in the center zone across different treatment groups during the light–dark alternating phase. “L” and “D” represent light and dark conditions, respectively. All data are expressed as mean ± standard deviation. ^#^, significant difference in time spent in the central zone between light and dark conditions within the same group (^#^, *p* < 0.05; ^##^, *p* < 0.01.) *, significant difference compared to the control group under the same light or dark condition (*, *p* < 0.05; **, *p* < 0.01; ***, *p* < 0.001). ^&^, significant difference compared to the BPA + GSK group under the same light or dark condition (^&^, *p* < 0.05; ^&&&^, *p* < 0.001).

Under continuous illumination, zebrafish larvae exposed to BPA exhibited a significant reduction in thigmotactic behavior compared to the control group (Figure 3A). While GSK (an NR1D1 agonist) alone did not significantly affect thigmotactic behavior, co-treatment with BPA and GSK (BPA + GSK group) mitigated BPA-induced reductions, restoring behavior to levels comparable to the control group.

During the light/dark cycle, thigmotactic behavior was significantly lower under dark conditions across all groups except the BPA + GSK group, which showed no significant differences between light and dark phases (Figure 3B). The results demonstrate that BPA exposure impairs thigmotactic behavior, a hallmark of anxiety-like behavior, and that NR1D1 activation can ameliorate these effects.

### 3.3. BPA Disrupts Spontaneous Locomotor Rhythms in Zebrafish Larvae

**Figure 4:**
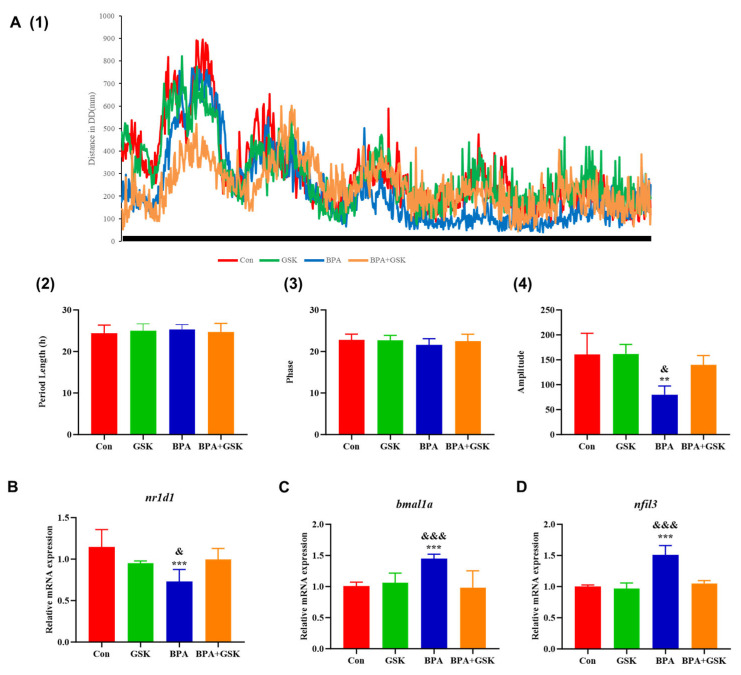
(**A**(1–4)) The effects of different treatments on the spontaneous activity rhythms of zebrafish larvae under constant dark (DD) conditions. (**B**–**D**) Effects of various treatments on expression of clock genes in zebrafish larvae. Data are presented as mean ± standard deviation. * indicates a significant difference from the control group, with **, *p* < 0.01; ***, *p* < 0.001. & indicates a significant difference from the BPA + GSK group, with ^&^, *p* < 0.05; ^&&&^, *p* < 0.001.

BPA exposure significantly reduced the amplitude of spontaneous locomotor rhythms in zebrafish larvae under constant darkness (DD) conditions (Figure 4A (1)). The motor period and phase remained constant in both the BPA and BPA+GSK groups when compared to the control group (Figure 4A (2–3)). However, the BPA + GSK group restored locomotor amplitude to levels comparable to the control group (Figure 4A (3)). The locomotor period and phase Transcriptomic analysis levels revealed a 36.2% reduction in *nr1d1* expression following BPA exposure, while its downstream target genes *bmal1a* and *nfil3* were up-regulated by 43.68% and 51.04%, respectively (Figure 4B–D). GSK treatment effectively normalized the expression of these genes.

### 3.4. BPA Exacerbates Astrocyte Activation in the Brain of Zebrafish Larvae

**Figure 5:**
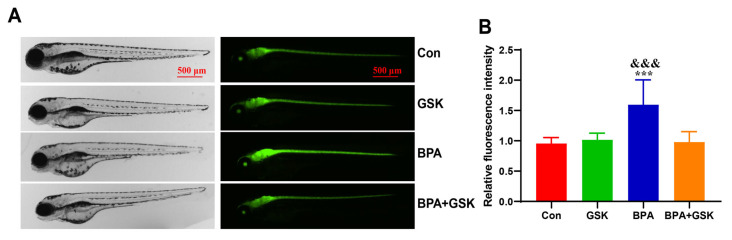
(**A**) Fluorescence images of Tg (gfap:EGFP) zebrafish larvae in different treatment groups. (**B**) Quantitative results of fluorescence intensity obtained from panel (**A**). Data are presented as mean ± standard deviation. * indicates a significant difference from the control group, with ***, *p* < 0.001. indicates a significant difference from the BPA + GSK group, with ^&&&^, *p* < 0.001.

To assess the effect of BPA on astrocyte activation, the fluorescence intensity was analyzed in Tg (gfap:EGFP) zebrafish larvae. BPA exposure resulted in a 67.07% increase in fluorescence intensity compared to the control group, indicating enhanced astrocyte activation (Figure 5A,B). However, no significant differences in fluorescence intensity were observed between the BPA + GSK, GSK and control groups. These results suggest that BPA induces astrocyte activation, contributing to the formation of an inflammatory microenvironment, which is mitigated by GSK treatment.

### 3.5. BPA Elevates Inflammatory Cytokine Levels

**Figure 6:**
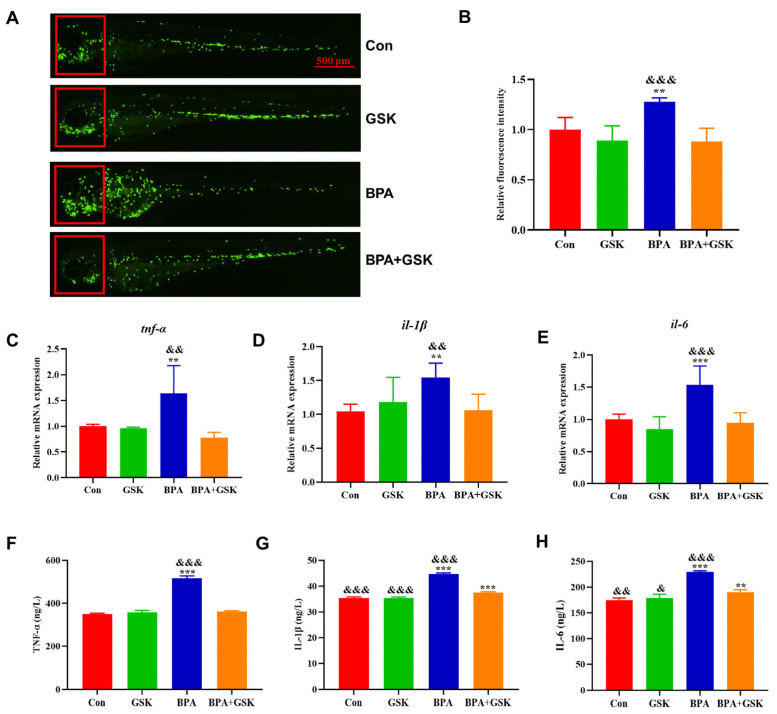
(**A**) Fluorescence images of Tg (lyz:EGFP) zebrafish larvae from different treatment groups, with the head region highlighted by a red box. (**B**) Semi-quantitative analysis of fluorescence intensity within the red box. (**C**–**E**) Effects of various treatments on the transcription levels of inflammatory cytokines (TNF-α, IL-1β, and IL-6) in zebrafish larvae. (**F**–**H**) Effects of different treatments on the levels of inflammatory cytokines (TNF-α, IL-1β, and IL-6) in zebrafish larvae. Data are presented as mean ± standard deviation. * indicates a significant difference from the control group, with **, *p* < 0.01; ***, *p* < 0.001. ^&^ indicates a significant difference from the BPA + GSK group, with ^&^, *p* < 0.05; ^&&^, *p* < 0.01; ^&&&^, *p* < 0.001.

BPA exposure significantly elevated the transcription and protein levels of inflammatory cytokines, including TNF-α, IL-1β, and IL-6, in zebrafish larvae. ELISA results showed increases of 63.74%, 48.43%, and 53.58%, respectively, compared to the control group. Co-treatment with GSK partially restored TNF-α, IL-1β, and IL-6 levels, though they remained slightly elevated compared to the control group (Figure 6C–H).

Fluorescence analysis of Tg (lyz:EGFP) zebrafish larvae revealed increased neutrophil migration to the head region following BPA exposure (27.88% increase), which was alleviated by GSK treatment (Figure 6A,B). These results indicate that BPA-induced inflammation is mediated by increased astrocyte activation and cytokine production, which can be partially reversed by NR1D1 activation.

### 3.6. BPA Disrupts Dopamine System in Zebrafish Larvae

**Figure 7:**
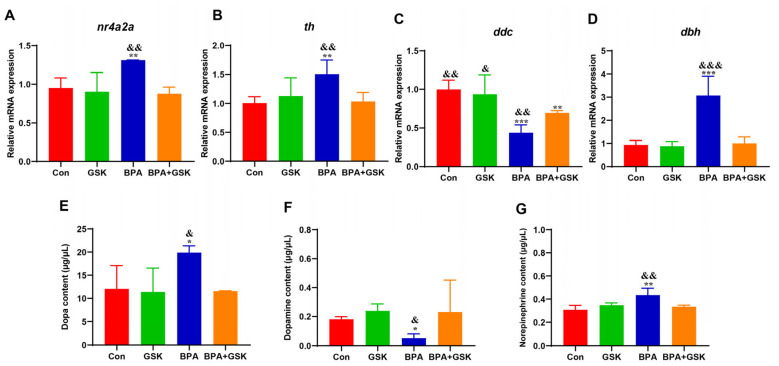
(**A**–**D**) Data are expressed as mean ± standard deviation. (**E**) Dopamine (DOPA); (**F**) Dopamine (DA); (**G**) Norepinephrine (NE). Data are expressed as mean ± standard deviation. * indicates a significant difference from the control group, with *, *p* < 0.05; **, *p* < 0.01; ***, *p* < 0.001. & indicates a significant difference from the BPA + GSK group, with ^&^, *p* < 0.05; ^&&^, *p* < 0.01; ^&&&^, *p* < 0.001.

BPA exposure significantly altered the transcription levels of genes involved in dopamine synthesis and metabolism. Specifically, BPA exposure increased *nr4a2a, th*, and *dbh* expression by 37.99%, 49.80%, and 228.87%, respectively, while decreasing *ddc* expression by 56%, 16% (Figure 7A–D). Co-treatment with GSK restored these gene expression levels to near-control levels.

Neurotransmitter analysis revealed that BPA exposure increased DOPA and norepinephrine (NE) levels while reducing dopamine (DA) levels (Figure 7E–G). GSK treatment effectively normalized these neurotransmitter levels. These findings suggest that BPA-induced dopamine dysregulation, characterized by impaired dopamine synthesis and metabolism, contributes to anxiety-like behavior and can be mitigated by NR1D1 activation.

## 4. Discussion

This study provides novel insights into the mechanistic basis of anxiety-like behaviors induced by early-life exposure to bisphenol A (BPA). Our findings demonstrate that BPA disrupts circadian rhythms, dopaminergic neuronal regulation, and inflammatory responses in zebrafish larvae, with NR1D1 playing a central role in these processes. Moreover, activation of NR1D1 by its agonist GSK effectively mitigated BPA-induced behavioral and molecular alterations, highlighting the therapeutic potential of targeting NR1D1 in anxiety disorders.

Circadian rhythms are essential for maintaining physiological and behavioral homeostasis, and their disruption has been implicated in the pathogenesis of anxiety disorders [23,24]. In this study, BPA exposure significantly suppressed the expression of *nr1d1*, a core circadian clock gene, while up-regulating *clocka* and other circadian regulators such as bmal1a and *nfil3*. These transcriptional changes were accompanied by a reduction in locomotor rhythmicity under DD conditions, a hallmark of circadian rhythm disruption.

The down-regulation of *nr1d1* observed in our study may lead to a dysregulated circadian clock, resulting in both behavioral abnormalities and systemic inflammation. Specifically, NR1D1 functions as a transcriptional repressor within the circadian clock system, regulating the expression of genes involved in metabolism, inflammation, and neurobehavioral processes [17]. Interestingly, GSK treatment restored *nr1d1* expression and rescued locomotor amplitude, suggesting that NR1D1 activation can counteract BPA-induced circadian misalignment.

Prior evidence supports the idea that environmental endocrine disruptors, including BPA, perturb circadian rhythms by interfering with the transcriptional activity of clock genes [11,25]. For example, experimental studies have shown that BPA exposure during pregnancy disrupts circadian gene expression, leading to alterations in behavior and neurological functions [26]. These findings emphasize the critical role of circadian rhythm integrity in modulating anxiety-like behaviors and support the hypothesis that NR1D1 is a key mediator of BPA toxicity.

The dopaminergic system plays a critical role in regulating mood, motivation, and anxiety-like behaviors [15,27]. Our transcriptomic analysis revealed that BPA exposure significantly up-regulated the expression of *nr4a2a*, *th*, and *dbh*, while down-regulating *ddc*, a key enzyme in dopamine synthesis. These molecular alterations were accompanied by increased DOPA and norepinephrine levels and decreased dopamine levels, suggesting that BPA disrupts dopamine homeostasis.

The present study reveals that BPA results in a reduction of dopamine (DA) levels in zebrafish larvae and the induction of anxiety-like behaviors, as evidenced by an increase in thigmotactic behavior. These findings are consistent with those of previous studies by Yu [28] and Wang [29], in which GSK was shown to effectively restore dopamine levels and attenuate anxiety-like behaviors. This evidence suggests that *nr1d1* plays a protective role in the dopamine system. It is hypothesized that BPA disrupts the synthesis and metabolism of dopamine by inhibiting *nr1d1* transcription, which in turn leads to the manifestation of anxiety-like behaviors in zebrafish larvae. BPA exposure has been demonstrated to result in a reduction in *nr1d1* transcription and an increase in nr4a2a levels. Consequently, this has been observed to result in an increase in tyrosine hydroxylase (*th*) transcriptional activity and DOPA levels. Further analysis of genes involved in dopamine production and metabolism showed that BPA significantly decreased the transcription of *ddc*, which is responsible for the conversion of DOPA to dopamine, and increased the transcription of *dbh*. This suggests that there is an increased conversion of dopamine to norepinephrine (NE), which ultimately results in a reduction in DA levels. In conclusion, while BPA increases DOPA levels by inhibiting *nr1d1* transcription, it decreases DA levels by impairing dopamine conversion and promoting its metabolism. It is noteworthy that GSK has the capacity to fully restore the transcriptional activity of genes involved in dopamine synthesis and metabolism, thereby normalizing levels of DOPA, dopamine, and norepinephrine. This finding suggests that nr1d1 may regulate the dopamine system via mechanisms that extend beyond its influence on th.

Mechanistically, BPA appears to inhibit *nr1d1* transcription, indirectly disrupting dopamine synthesis and metabolism. Elevated *nr4a2a* levels may reflect a compensatory response to neuronal dysfunction, consistent with its role in dopaminergic neuron differentiation and survival [30]. Furthermore, BPA-induced reductions in dopamine levels are likely due to impaired *ddc* expression, which is responsible for converting DOPA to dopamine, and increased *dbh* expression, which promotes dopamine conversion to norepinephrine. These disruptions in dopamine metabolism align with prior studies reporting BPA-induced alterations in dopaminergic signaling, leading to anxiety-like behaviors [28,29]. Interestingly, GSK treatment normalized both neurotransmitter levels and the expression of dopaminergic regulatory genes, indicating that *nr1d1* activation can mitigate BPA-induced dopaminergic dysregulation. This finding underscores the interplay between circadian regulation and the dopaminergic system in mediating anxiety-like behaviors.

Our study demonstrated that BPA exposure enhances astrocyte activation and induces a pro-inflammatory microenvironment, as evidenced by increased expression of inflammatory cytokines (TNF-α, IL-1β, and IL-6) and neutrophil migration. BPA-induced astrocyte activation, observed in Tg (gfap:EGFP) zebrafish, is consistent with previous studies linking environmental toxins to neuroinflammation [31]. Astrocytes, while essential for CNS homeostasis under normal conditions, can exacerbate neuroinflammation and contribute to anxiety-like behaviors when overactivated [32].

Kunz et al. reported that BPA exposure during pregnancy and lactation increased astrocyte activation, similar to the findings in our study [33]. Moreover, BPA exposure elevated the transcriptional and protein levels of inflammatory cytokines, promoting neutrophil migration to the head region in Tg (lyz: EGFP) zebrafish larvae. These results suggest that BPA-induced inflammation is mediated by astrocyte activation and innate immune responses. Notably, GSK treatment significantly reduced astrocyte activation, inflammatory cytokine levels, and neutrophil migration, highlighting NR1D1’s anti-inflammatory role in CNS inflammation [34,35].

NR1D1 is a nuclear receptor which is central to the circadian clock system. This regulates genes linked to metabolism, inflammation, and neuronal function. We have found strong evidence that NR1D1 is key to BPA-induced toxicity. BPA significantly down-regulates NR1D1 expression, disrupting rhythms. BPA can also interfere with the activity of NR1D1 via interactions with estrogen and androgen receptors. Bioinformatics using JASPAR database analysis shows the binding site of ESRAR on the NR1D1 promoter [36]. This suggests that BPA directly targets the clock system, affecting physiology and behavior.

This study provides valuable insights into the mechanisms underlying BPA-induced anxiety-like behaviors. Nevertheless, the study is not without its limitations. While zebrafish larvae have proven to be a valuable model system, it must be acknowledged that they are not devoid of limitations, particularly with regard to fully capturing the intricacies of mammalian neurobiology [31,37]. During the five-day monitoring period, significant variations in zebrafish larvae distances were observed on the first day. However, by the final day, the depletion of food resources and the deterioration of the aquatic environment resulted in a tendency towards negative larval activity levels, with distances being minimal or static across all groups. Consequently, the data set was limited to the middle three days, which were found to be relatively stable. Another limitation is the use of only one behavioral test to assess anxiety-like responses in zebrafish larvae [38]. Moreover, while GSK4112 is a well-characterized NR1D1 agonist [39], it is imperative that its potential off-target effects on other nuclear receptors (NRs) are meticulously evaluated. This finding indicates a significant direction for future research endeavors. The present study is part of a broader program of research, the aim of which is to establish the necessary experimental systems to address this issue in subsequent studies.

Consequently, it is essential for future studies to validate these findings using mammalian models. The present study has identified NR1D1 and the precise molecular mechanisms by which BPA disrupts NR1D1 activity, and these require further investigation. The therapeutic potential of NR1D1 activation warrants further exploration in clinical settings.

## 5. Conclusions

**Figure 8:**
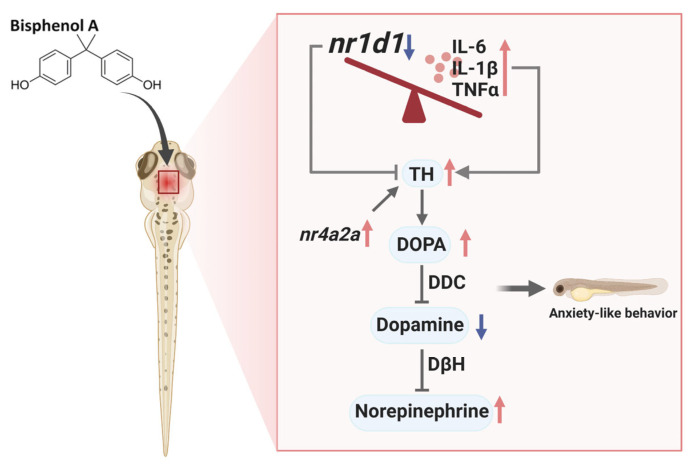
The mechanism of core clock gene nr1d1 in anxiety-like behavior induced by BPA in zebrafish larvae. Red arrows represent increasing levels; black coloured arrows represent decreasing levels.

In summary, the present study investigated the effects of early exposure to BPA on anxiety-like behavior and its underlying mechanisms in zebrafish larvae. The findings suggest that exposure to BPA induces anxiety-like behaviors, accompanied by cellular activation and brain inflammation. Mechanistic exploration revealed that BPA suppresses the expression of the core circadian clock gene nr1d1, which in turn negatively regulates inflammatory factors. Consequently, exposure to BPA has been demonstrated to elevate transcript and protein levels of IL-6, IL-1β, and TNF-α, resulting in an exacerbation of the inflammatory microenvironment and disruption of the balance of the dopamine system. This disruption has been shown to contribute significantly to the development of anxiety-like behavior in zebrafish larvae (Figure 8). The findings emphasize the pivotal function of NR1D1 in mediating the neurotoxic effects of BPA, thereby establishing a connection between circadian dysregulation, neuroinflammation, dopaminergic dysfunction, and anxiety-like behaviors. This study provides novel insights into the mechanisms by which BPA induces neurodevelopmental toxicity and highlights NR1D1 as a potential therapeutic target to mitigate the effects of early BPA exposure.

## Data Availability

All data generated or analyzed during this study are included in this published article (and its Supplementary Information Files).

## Supplementary Materials

The following supporting information can be downloaded at: https://www.mdpi.com/article/10.3390/toxics13060449/s1, Figure S1: Primer sequences; Table S1: The number of differentially expressed genes with increased or decreased abundance.
