# Supplementary material for: Role of NR1D1 in Bisphenol A-Induced Anxiety-like Behavior and Inflammation in Zebrafish Larvae
Source: Toxics. 2025 May 28;13(6):449. doi: 10.3390/toxics13060449 (PMC12197085; doi:10.3390/toxics13060449)
Supplement: Supplementary file 1 [file toxics-13-00449-s001.zip › toxics-3586155-supplementary.pdf]

The roles and mechanisms of *nr1d1* in anxiety-like behavior induced by BPA in zebrafish larvae

Supplementary Material

Table S1. Primer sequences

Name	Primer type	Primer sequence（5'-3'）
β-actin		ACGAACGACCAACCTAAACTCT
		TTAGACAACTACCTCCCTTTGC
nr1d1		ACATCCCAGGGTTTAGCACG
		GTTCGGAGAGCCCGGATTAG
bmal1a		GAAGACATTACGAGGGGCCA
		AGAGGAAACCATCAGCAGCC
clocka		CGAAACGGCTTTGAGGGAGT
		CAAACGGCAGGTAACCAATGAT
nfil3		ATCACCAGGAGGCCCTAACT
		CTTTTCAAGCAGGCCACTTC
nr4a2a		CAGGTCCAACCCGATGGAAA
		TCCGTGTCTCTCTGTGACCA
ddc		CCGCAAGCATGTAGGACTG
		GCTGTTGATCCTCTTCAGCAG=
tnf-α		GCGCTTTTCTGAATCCTACG
		TGCCCAGTCTGTCTCCTTCT
il-1β		GGCTGTGTGTTTGGGAATCT
		TGATAAACCAACCGGGACA
il-6		GCGTCCTGACGTGGTATAAAG
		GTCGTTTGGTGCTGTGTTTG
th		CAAGCAGCTCCACATCTTCC
		CATCGCTCTCCTCAAACACG
dbh		TGCAACCAGTCCACAGCGCA
		GCTGTCCGCTCGCACCTCTG

Figure S1

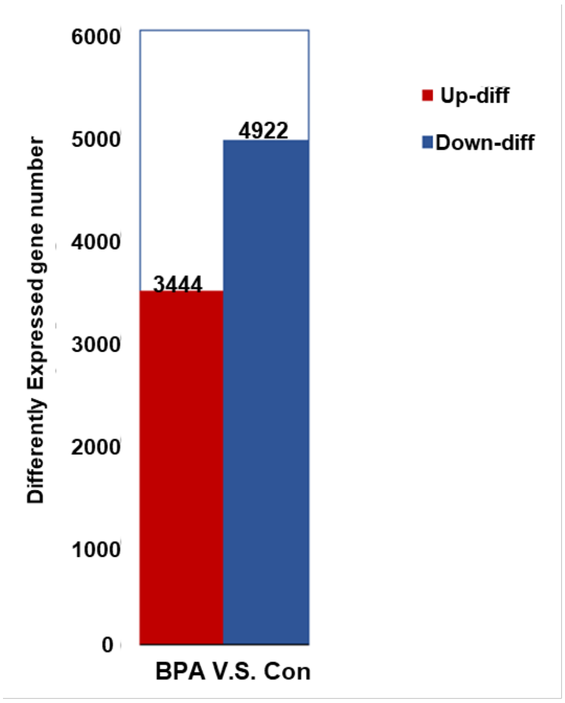

Figure S1. The number of differentially expressed genes with increased or decreased abundance.
